# Supplementary material for: A pilot study of closed-loop neuromodulation for treatment-resistant post-traumatic stress disorder
Source: Nat Commun. 2023 May 24;14:2997. doi: 10.1038/s41467-023-38712-1 (PMC10209131; doi:10.1038/s41467-023-38712-1)
Supplement: Supplementary file 1 — Supplementary Information [file 41467_2023_38712_MOESM1_ESM.pdf]

Online Materials

Supplementary Tables and Figures

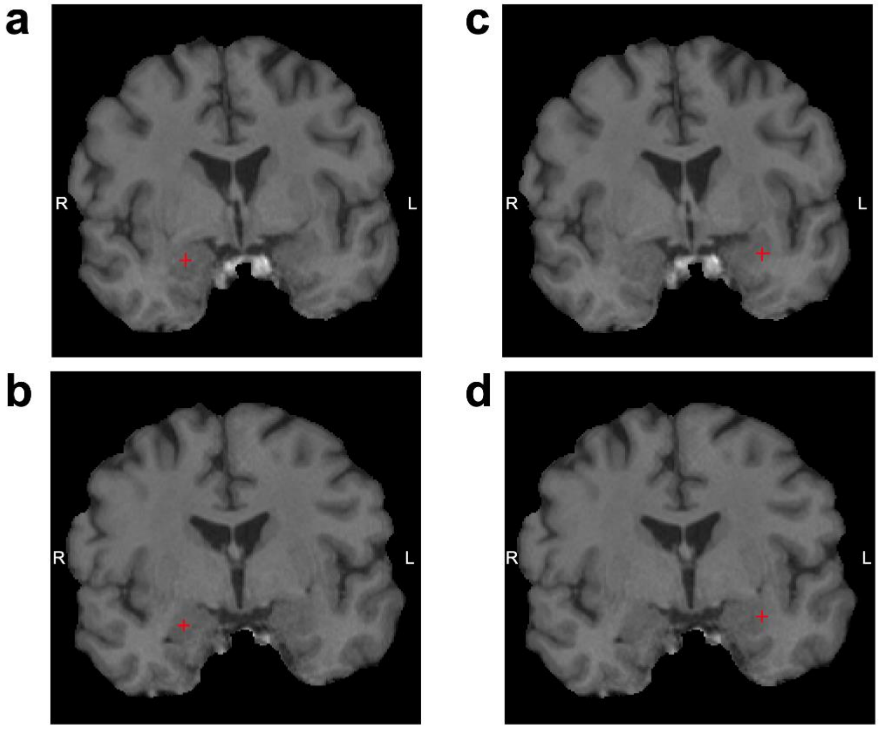

Supplementary Figure 1. TR-PTSD 1 electrode contact localizations. RNS contacts localized in the amygdala that comprise right (**a-b**) and left (**c-d**) recording channels in TR-PTSD 1 visualized in participant's anatomical MRI space. **a** and **c** = anterior contacts; **b** and **d** = posterior contacts, R = right, L = left.

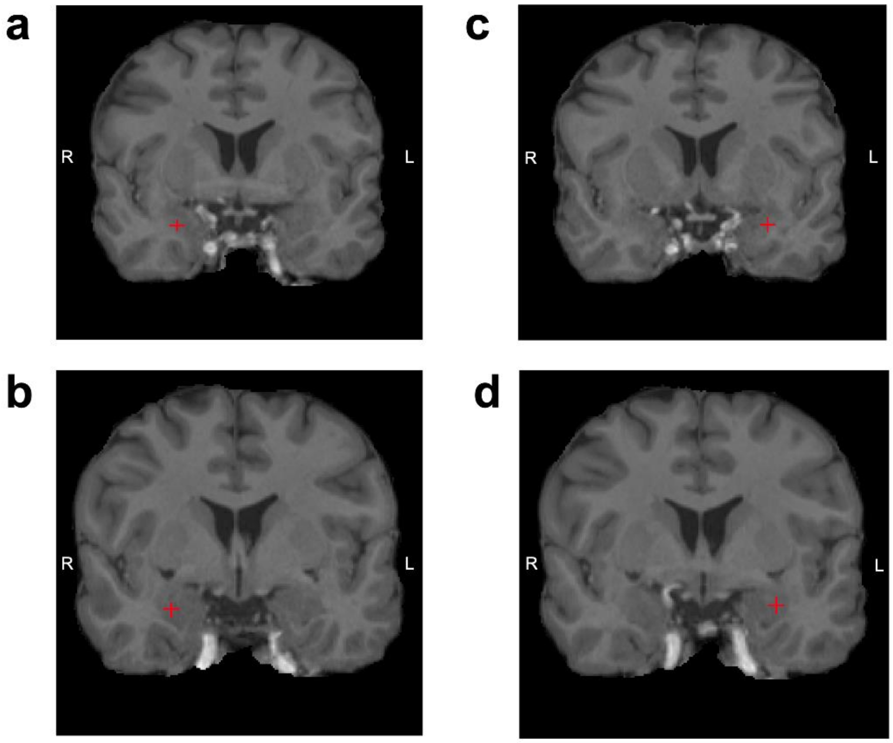

Supplementary Figure 2. TR-PTSD 2 electrode contact localizations. RNS contacts localized in the amygdala that comprise right (**a-b**) and left (**c-d**) recording channels in TR-PTSD 2 visualized in participant's anatomical MRI space. **a** and **c** = anterior contacts; **b** and **d** = posterior contacts, R = right, L = left.

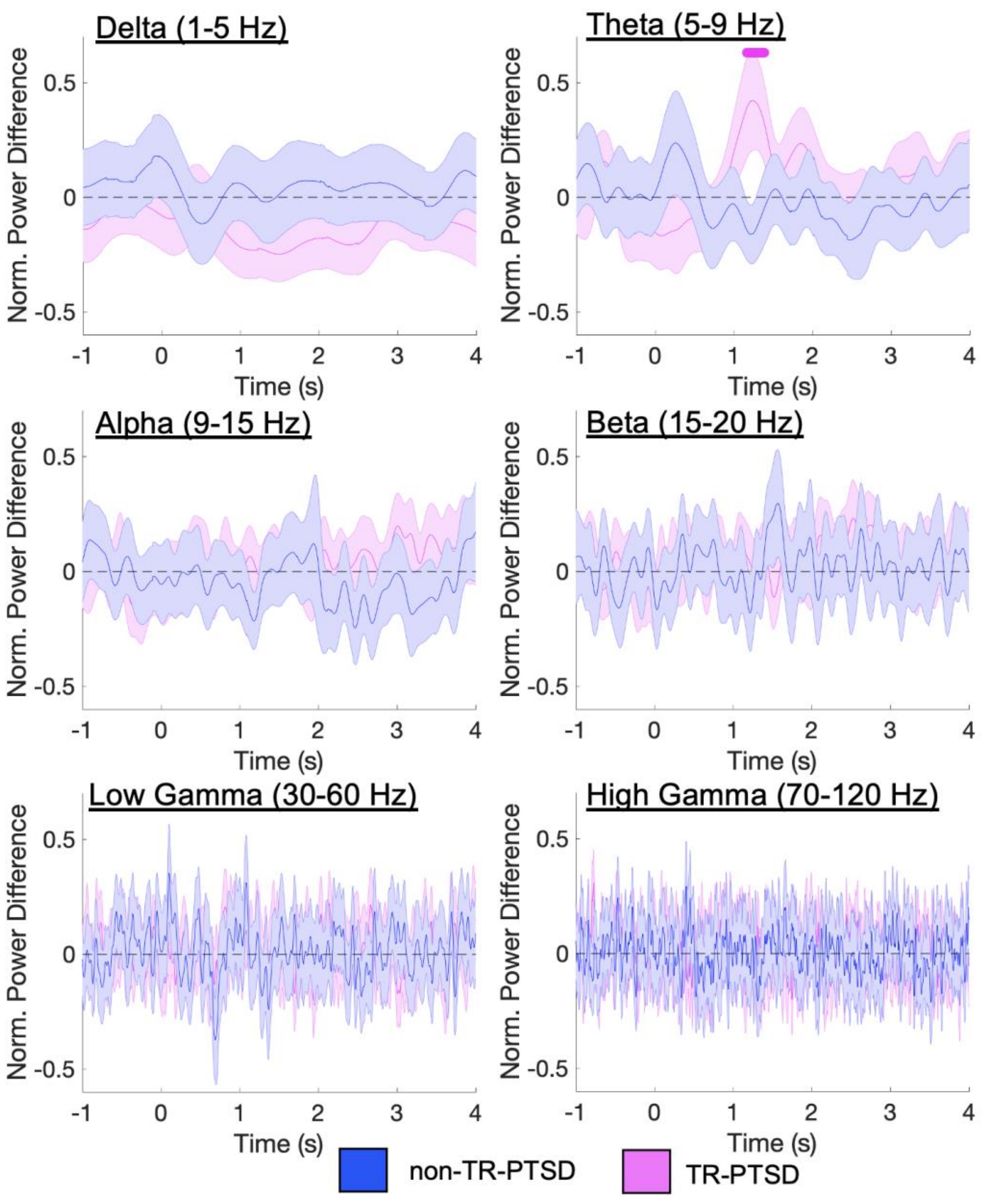

Supplementary Figure 3. Amygdala valence-related oscillatory changes in TR-PTSD are specific to the theta band. Normalized (Norm.) difference in frequency bandpower (delta, theta, alpha, beta, low and high gamma) between negative and positive/neutral conditions (negative – positive/neutral) during the Emotional Image Task (related to Fig. 2a) over time separately for TR-PTSD (magenta) and non-TR-PTSD (blue) participants relative to image onset (time 0). Positive norm. power difference = negative > positive/neutral; negative norm. power difference = positive/neutral > negative. For each frequency band, the mean ± 2 s.e.m. (standard error of the mean) across channels is shown for TR-PTSD ($N_{participants}$ = 2, $N_{channels}$ = 4) and non-TR-PTSD ($N_{participants}$ = 6, $N_{channels}$ = 9) participants. Horizontal bars indicate time points where there were significant differences, which occurred only in theta bandpower (5-9 Hz) between negative and positive/neutral trials in TR-PTSD (magenta) but not non-TR-PTSD (blue) participants ($p < 0.05$, Bonferroni corrected) using linear mixed effects models (Methods).

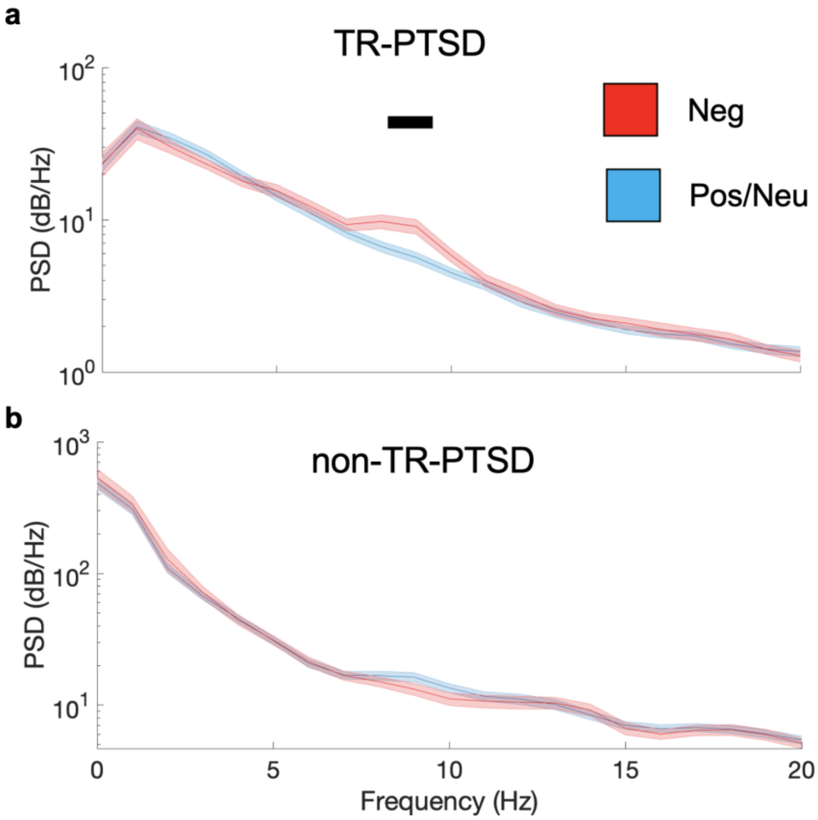

Supplementary Figure 4. Negative-valence-related amygdala changes only in TR-PTSD participants. a, Mean ± s.e.m. power spectral density (PSD, dB/Hz) during negative (red) and positive/neutral images (blue) at specified frequency steps (1–20 Hz) for TR-PTSD participants ($N_{participants}$ = 2, $N_{channels}$ = 4) during the pre-stimulation period. Black bar = $p < 0.05$, linear mixed model, Bonferroni corrected. **b**, Mean ± s.e.m. PSD in non-TR-PTSD participants ($N_{participants}$ = 6, $N_{channels}$ = 9). during negative (red) and positive/neutral images (blue) at specified frequency steps (1–20 Hz). Significance ($p < 0.05$, linear mixed model, Bonferroni corrected) not found.

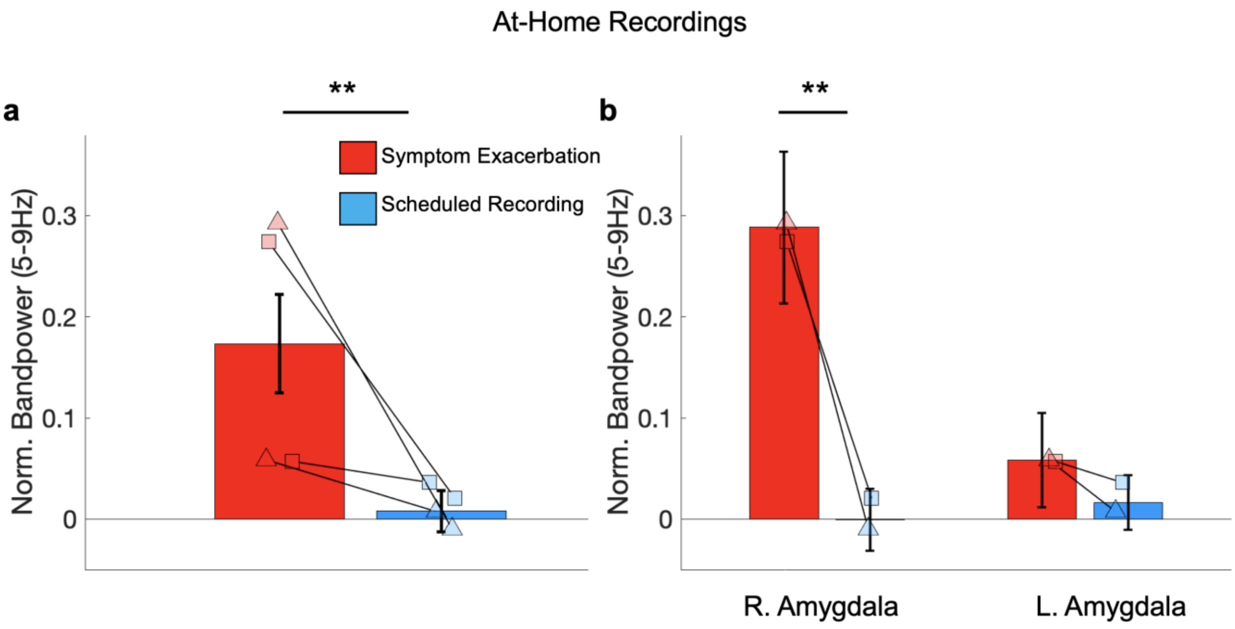

Supplementary Figure 5. Increased amygdala theta activity during PTSD-related symptomatic episodes. a. Normalized (Norm.) mean ± s.e.m. theta (5-9 Hz) bandpower within the amygdala of TR-PTSD participants during symptomatic (red) and asymptomatic (blue) periods (For each bar $N_{participants} = 2$, $N_{channels} = 4$ (left and right amygdala), squares = TR-PTSD 1, triangles = TR-PTSD 2) . ** = unadjusted p = 0.007 using linear mixed model (Methods). **b**, Same as **a**, but separated by hemisphere (R = Right, L = Left; For each bar $N_{participants} = 2$, $N_{channels} = 2$, squares = TR-PTSD 1, triangles = TR-PTSD 2). ** = unadjusted p = 0.002 using linear mixed model (Methods).

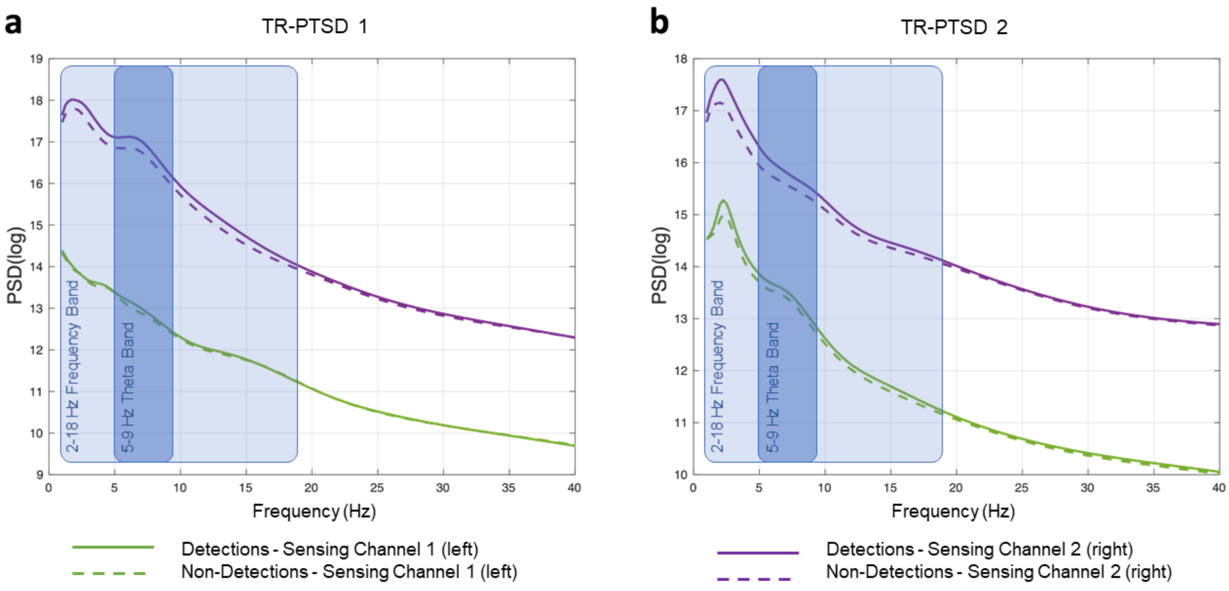

Supplementary Figure 6. Increased low frequency activity during stimulation-triggering detections. Power spectral density (PSD) for TR-PTSD 1 (**a**) and TR-PTSD 2 (**b**) during Trigger 1 (Suppl. Table 5) demonstrating elevations in 2-18 Hz bandpower (light blue shaded areas) and 5-9 Hz theta power (dark blue shaded areas) in left (green) and right (purple) sensing channels during detection (solid curve) periods compared to non-detection (dashed curve) periods.

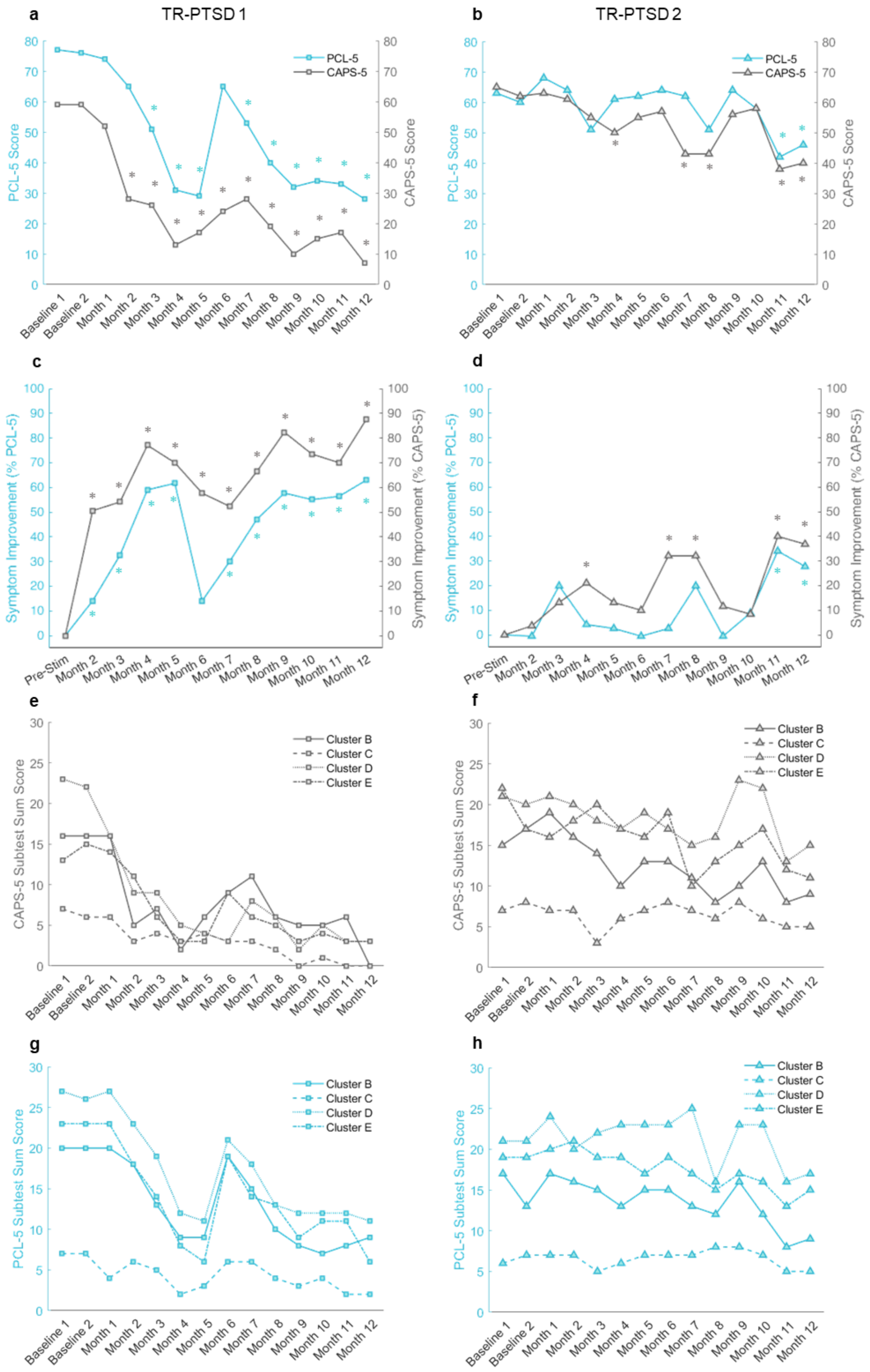

a
TR-PTSD 1
PCL-5
CAPS-5
PCL-5 Score
CAPS-5 Score
b
TR-PTSD 2
PCL-5
CAPS-5
PCL-5 Score
CAPS-5 Score
Baseline 1
Baseline 2
Month 1
Month 2
Month 3
Month 4
Month 5
Month 6
Month 7
Month 8
Month 9
Month 10
Month 11
Month 12
c
Symptom Improvement (% PCL-5)
Symptom Improvement (% CAPS-5)
Pre-Stim
d
Symptom Improvement (% PCL-5)
Symptom Improvement (% CAPS-5)
e
CAPS-5 Subtest Sum Score
Cluster B
Cluster C
Cluster D
Cluster E
f
CAPS-5 Subtest Sum Score
Cluster B
Cluster C
Cluster D
Cluster E
g
PCL-5 Subtest Sum Score
Cluster B
Cluster C
Cluster D
Cluster E
h
PCL-5 Subtest Sum Score
Cluster B
Cluster C
Cluster D
Cluster E

Supplementary Figure 7. CAPS-5 and PCL-5 subtest scores over time. Changes in CAPS-5 scores (gray) and PCL-5 scores (cyan) for TR-PTSD 1 (**a**) and TR-PTSD 2 (**b**) during Pre-Stim periods (baseline 1, 2, and month 1) and Post-Stim periods (months 2-12). Gray/cyan * = reliable change in CAPS-5 or PCL-5 scores, respectively, according to threshold for sample 1 reported in Marx et al.[1] relative to pre-stimulation baseline assessments (mean of baseline 1, 2, and month 1). **c-d:** % Symptom improvement on CAPS-5 (gray) and PCL-5 (cyan) during Pre-Stim periods (baseline 1, 2, and month1) and Post-Stim periods (months 2-12). Gray/cyan * = reliable change in CAPS-5 or PCL-5 scores, respectively, according to threshold for sample 1 reported in Marx et al.10 relative to pre-stimulation baseline assessments (mean of baseline 1, 2, and month 1). **e-h:** CAPS-5 (gray) and PCL-5 (cyan) subtests including Cluster B (intrusive symptoms), Cluster C (avoidance), Cluster D (negative alterations to mood and cognition), and Cluster E (alterations in arousal and reactivity) over the Pre-Stim and Post-Stim periods for TR-PTSD 1 and TR-PTSD 2.

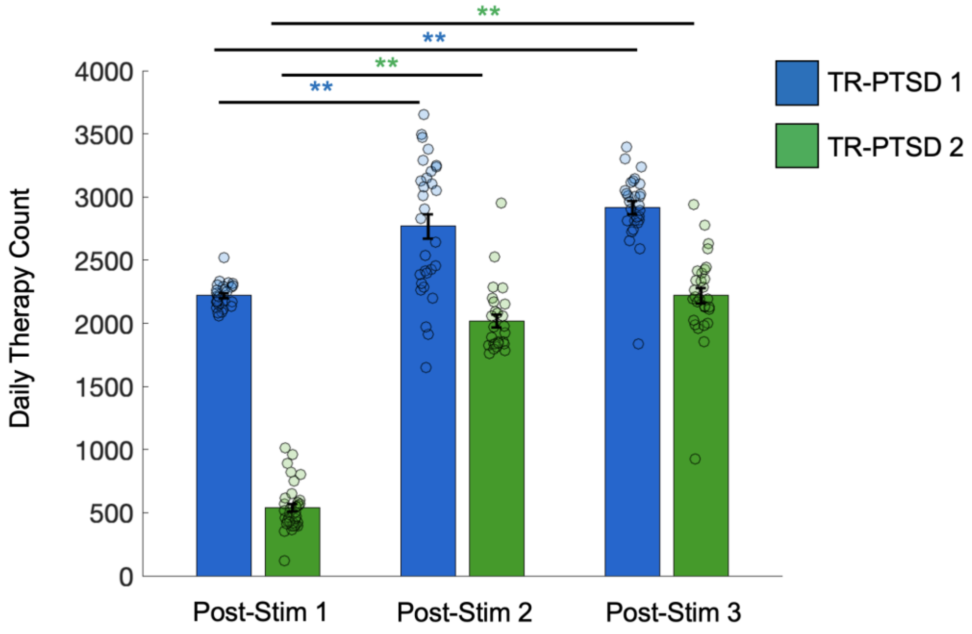

Supplementary Figure 8. Daily stimulation (therapy) counts in TR-PTSD. Mean (± s.e.m.) number of daily stimulation therapies delivered prior to Post-Stim 1 (months 2-3), Post-Stim 2 (months 5-6), and Post-Stim 3 (months 10-11) of closed-loop neuromodulation in TR-PTSD 1 (blue) and TR-PTSD 2 (green). TR-PTSD 1: Post-Stim 1 $N_{\text{therapy counts}}$ = 26, Post-Stim 2 $N_{\text{therapy counts}}$ = 30, Post-Stim 3 $N_{\text{therapy counts}}$ = 28; TR-PTSD 2: Post-Stim 1 $N_{\text{therapy counts}}$ = 35, Post-Stim 2 $N_{\text{therapy counts}}$ = 27, Post-Stim 3 $N_{\text{therapy counts}}$ = 30. ** = $p < 0.001$, Wilcoxon rank sum test, Bonferroni corrected.

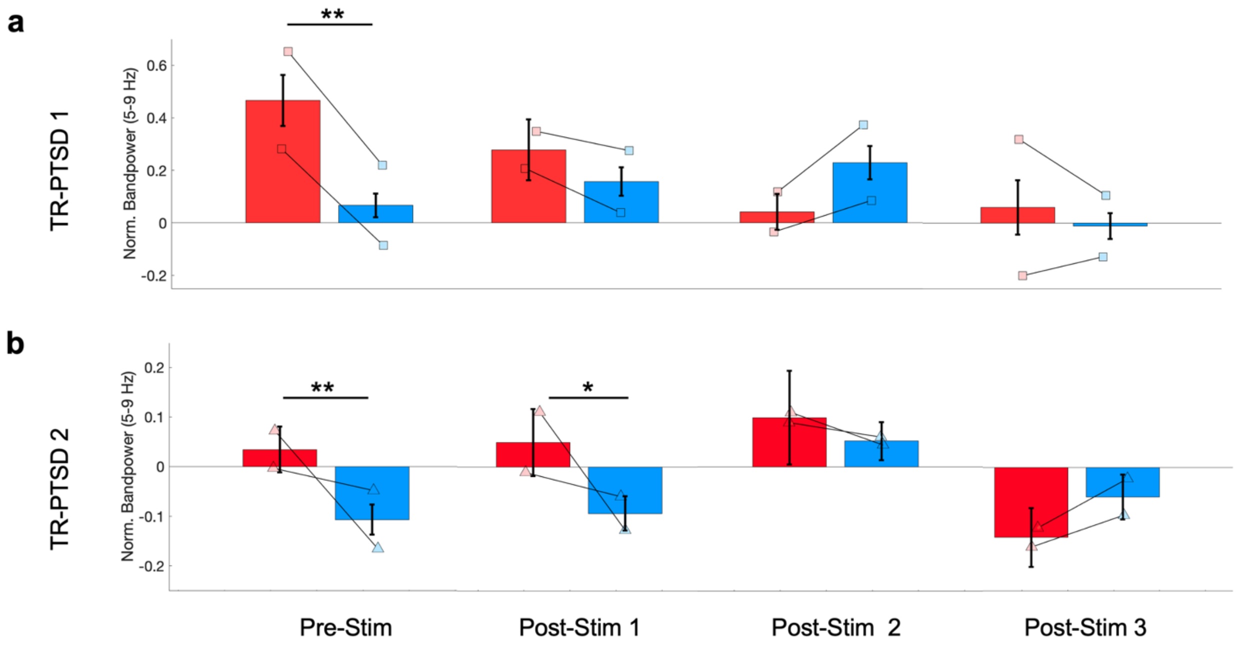

Supplementary Figure 9. Reduction in negative-image-related amygdala theta following stimulation. a, Normalized (Norm.) mean ± s.e.m. amygdala theta bandpower during negative (red) and positive/neutral (blue) images in TR-PTSD 1 (squares) and TR-PTSD 2 (triangles) during Pre-Stim and Post-Stim 1-3 (Fig. 1a) of the Emotional Image Task. For each bar $N_{participants} = 1$, $N_{channels} = 2$ (left and right amygdala). ** = unadjusted $p < 0.001$ using linear mixed model (Methods). **b**, Same as **a**, but for TR-PTSD 2. For each bar $N_{participants} = 1$, $N_{channels} = 2$ (left and right amygdala). Pre-Stim * = unadjusted $p = 0.008$, Post-Stim 1 * = unadjusted $p = 0.04$, using linear mixed model (Methods).

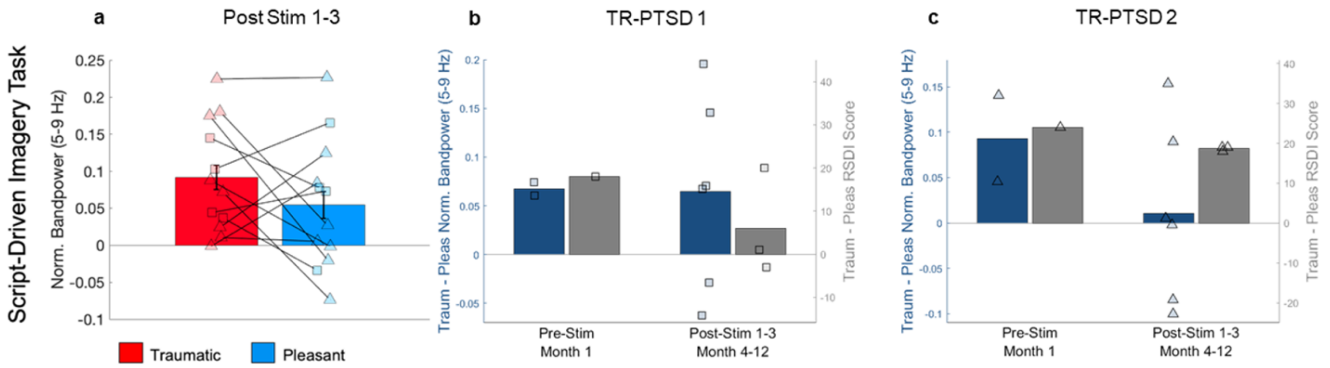

Supplementary Figure 10. Reduction in trauma-related amygdala theta following stimulation during the Script-Driven Imagery Task over all Post-Stim sessions and in relation to changes in state PTSD symptoms. a, Normalized (Norm.) mean ± s.e.m. amygdala theta bandpower during traumatic (red) and pleasant (blue) audio scripts in TR-PTSD 1 (squares) and TR-PTSD 2 (triangles) during Post-Stim 1-3 (Fig. 1a) combined due to limited trials obtained within each session (4 trials per condition). Each bar contains 12 data points ($N_{participants} = 2$, $N_{channels} = 2$ (left and right amygdala), $N_{sessions} = 3$). **b**, Difference in normalized (Norm.) amygdala theta (5-9 Hz) bandpower between traumatic (Traum) and pleasant (Pleas) audio scripts from the Emotional Image Task (navy) and RSDI scores (gray) for TR-PTSD 1 (squares) during Pre-Stim and Post-Stim 1-3. **c**, Same as **b** but for TR-PTSD 2 (triangles).

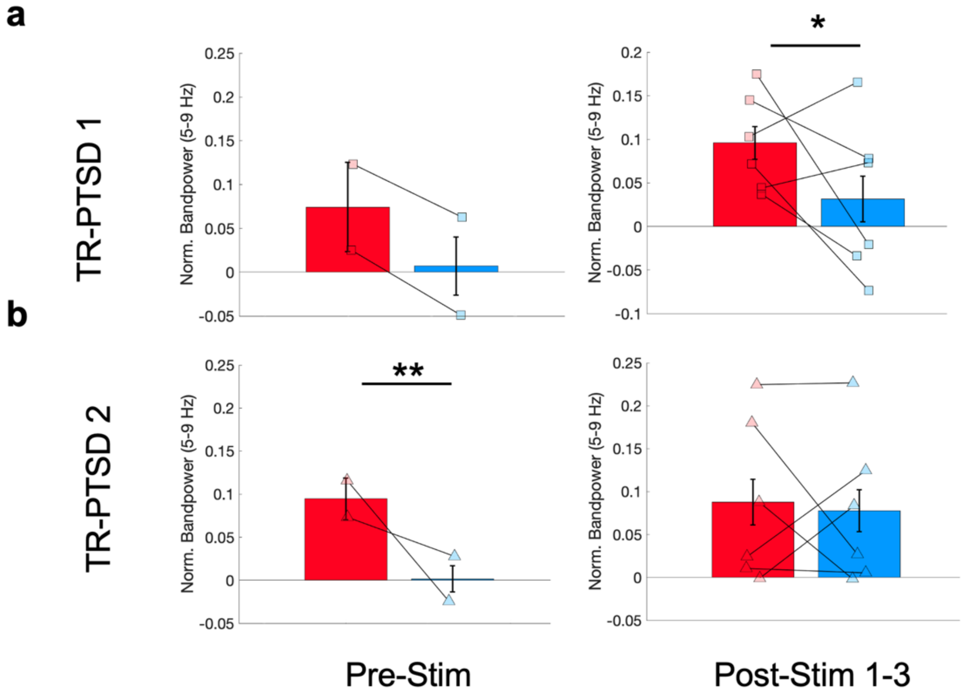

Supplementary Figure 11. Changes in trauma-related amygdala theta following stimulation. a, Normalized (Norm.) mean ± s.e.m. amygdala theta bandpower during traumatic (red) and pleasant (blue) audio scripts in TR-PTSD 1 (squares) during Pre-Stim and Post-Stim sessions 1-3 (Fig. 1a) combined due to limited trials obtained within each session (4 trials per condition). Pre-Stim (Left) $N_{participants} = 1$, $N_{channels} = 2$, $N_{sessions} = 1$. Post-Stim 1-3 (Right) $N_{participants} = 1$, $N_{channels} = 2$, $N_{sessions} = 3$. * = unadjusted $p = 0.04$, using linear mixed model (Methods). **b**, Same as **a** but in TR-PTSD 2. Pre-Stim (Left) $N_{participants} = 1$, $N_{channels} = 2$, $N_{sessions} = 1$. Post-Stim 1-3 (Right) $N_{participants} = 1$, $N_{channels} = 2$, $N_{sessions} = 3$. ** = unadjusted $p = 0.0057$, using linear mixed model (Methods).

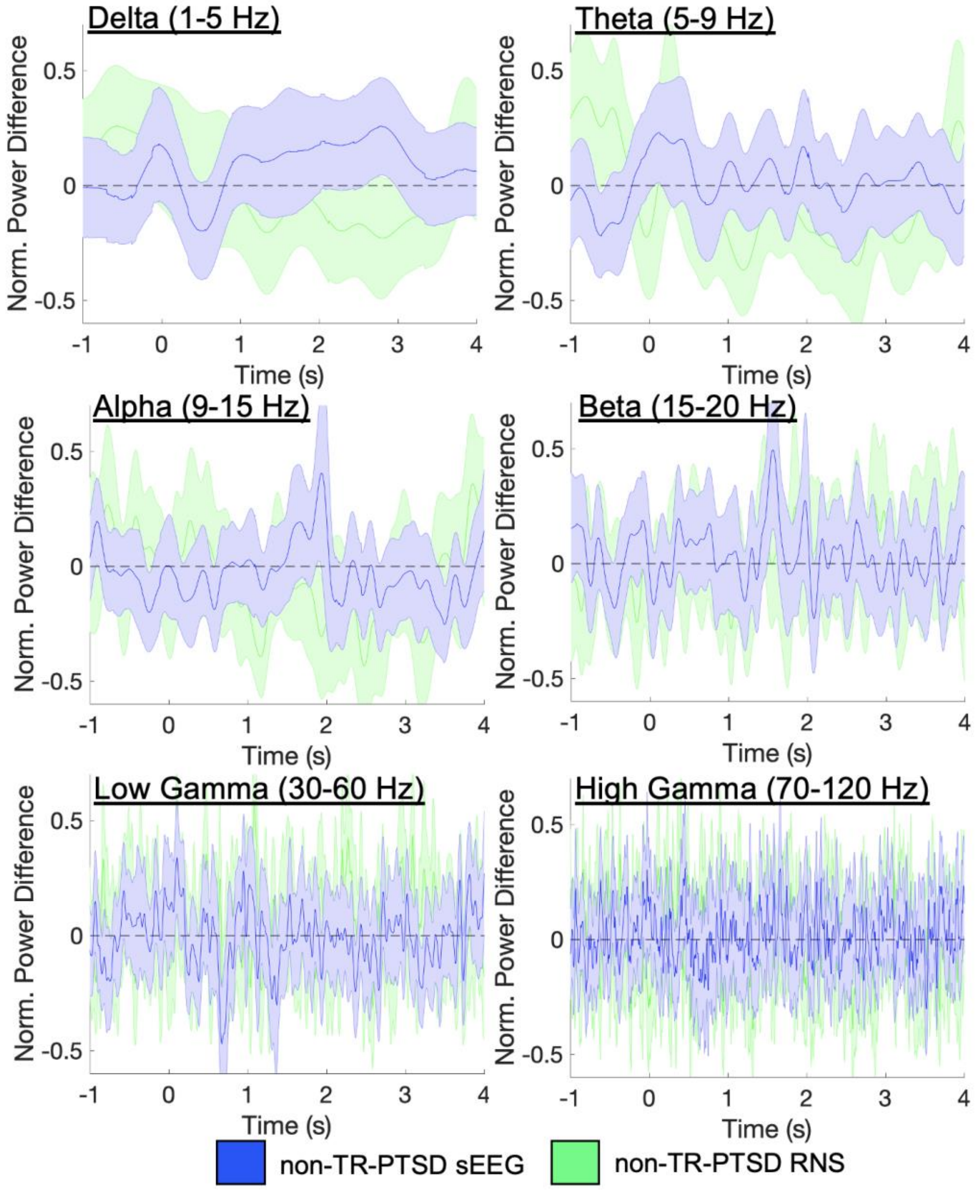

Supplementary Figure 12. Lack of amygdala valence-related changes in sEEG and RNS non-TR-PTSD participants. Normalized (Norm.) difference in frequency bandpower (delta, theta, alpha, beta, low and high gamma) between negative and positive/neutral conditions (negative – positive/neutral) during the Emotional Image Task (related to Fig. 2a) over time separately for sEEG (blue) and RNS (green) non-TR-PTSD participants relative to image onset (time 0). Positive norm. power difference = negative > positive/neutral; negative norm. power difference = positive/neutral > negative. For each frequency band, the mean ± 2 s.e.m. across channels is shown for non-TR-PTSD sEEG ($N_{participants}$ = 3, $N_{channels}$ = 3) and non-TR-PTSD RNS ($N_{participants}$ = 3, $N_{channels}$ = 6) participants. Significant valence-related changes using linear mixed effects models (see Supplemental Appendix) were not present in non-TR-PTSD, irrespective of recording modality.

Participant	Age (± s.e.m)	Gender	Handedness	Implanted System	Clinical Diagnoses that led to Electrode Implantation
TR-PTSD 1 and 2	38 ± 2	M	Ambidextrous	RNS-320	TR-PTSD
non-TR-PTSD 1	44	M	R	sEEG	Epilepsy
non-TR-PTSD 2	35	M	L	sEEG	Epilepsy
non-TR-PTSD 3	40	M	R	sEEG	Epilepsy
non-TR-PTSD 4	68	M	R	RNS-320	Epilepsy
non-TR-PTSD 5	28	M	R	RNS-320	Epilepsy
non-TR-PTSD 6	36	M	R	RNS-300M	Epilepsy

Supplementary Table 1. Participant demographics, implanted electrode systems, and treatment-resistant clinical diagnoses that led to electrode implantation. (sEEG = stereoelectroencephalography, NeuroPace RNS-320 and RNS-300M Systems). TR-PTSD = treatment-resistant post-traumatic stress disorder.

Participant	Left Amygdala	Right Amygdala
non-TR-PTSD 1	X	X
non-TR-PTSD 2		X
non-TR-PTSD 3		X
non-TR-PTSD 4	X	
non-TR-PTSD 5		X
non-TR-PTSD 6	X	

Supplementary Table 2. Electrode localizations for each epilepsy (non-TR-PTSD) participant showing whether each had a left and/or right amygdala channel that was included in the study.

Participant	Right<br>Amygdala<br>C1 (x,y,z)	Right<br>Amygdala<br>C2 (x,y,z)	Left<br>Amygdala<br>C1 (x,y,z)	Left<br>Amygdala<br>C2 (x,y,z)
TR-PTSD 1	(35,64,26)	(35,61,26)	(60,64,26)	(60,62,28)
TR-PTSD 2	(34,64,27)	(34,62,28)	(59,65,28)	(59,62,29)
non-TR-PTSD 1*	(37,62,25)<br>(32,62,25)	(35,62,25)<br>(30,62,25)	(55,63,25)<br>(59,63,25)	(57,63,25)<br>(62,63.25)
non-TR-PTSD 2	(34,62,27)	(31,62,27)		
non-TR-PTSD 3	(29,62,29)	(29,62,29)		
non-TR-PTSD 4			(56,63,30)	(57,61,30)
non-TR-PTSD 5	(32,65,23)	(32,61,25)		
non-TR-PTSD 6			(58,62,26)	(58,66,24)

Supplementary Table 3. Electrode localizations showing specific MNI coordinates (x,y,z) of both contacts (C1 and 2) of the left and right amygdala bipolar channels in each TR-PTSD participant that were used for iEEG recording and closed-loop stimulation. *Non-TR-PTSD 1 had two bipolar channels located within the amygdala bilaterally. MNI152_T1_2mm was used to register all images.

**Participant**	**Event**	**Symptom Description**	**SUDS Score**
TR-PTSD 1	1°	"Fireworks around neighborhood. Hypervigilant racy feeling."	8
TR-PTSD 1	2°	"Saw a very gory scene flash in my mind, weak knees, cold sweat, shaking, vision."	Not reported
TR-PTSD 2	1°	"Sad, depressed"	5
TR-PTSD 2	2°	"Angry, irritable, somewhat suicidal thoughts"	4-5

Supplementary Table 4. Examples of self-reported descriptions related to self-triggered recordings of symptom exacerbations (magnet swipe recordings) for TR-PTSD 1 and TR-PTSD 2. °These events triggered intrusive recollections of combat trauma. SUDS = Subjective Units of Distress Scale

Participant	Stimulation Logic	Proportion of Detection
TR-PTSD 1 (Month 1-12)	Trigger 1 or Trigger 2<br>(either hemisphere)	96% (Trigger 1) 4% (Trigger 2)
TR-PTSD 2 (Month 1-2)	Trigger 1 or Trigger 2<br>(both hemispheres)	51% (Trigger 1) 49% (Trigger 2)
TR-PTSD 2 (Month 3-12)	Trigger 1 or Trigger 2<br>(either hemisphere)	85% (Trigger 1) 15% (Trigger 2)

Supplementary Table 5. **RNS programming logic used in closed-loop stimulation.** Trigger 1: Greater than 50% change in area under the curve for low frequencies (2-18 Hz) for longer than 0.26 seconds in either posterior amygdala channel. Trigger 2: Greater than 62.5% change in average line length in either left or right posterior amygdala channel. In all cases, activation of stimulation trigger led to delivery of 100 ms of 1.0-3.0 mA current (charge-balanced biphasic square wave pulses) at 200 Hz with a pulse width of 160 microseconds delivered bilaterally, simultaneously to amygdala contacts (estimated charge density: 1.0 $\mu C/cm^2$). Daily therapy limit: 5000 stimulations.

Negative	Valence	Arousal	Dominance
TR-PTSD pre-stim	2.33 (0.08)	5.89 (0.11)	3.62 (0.10)
TR-PTSD post-stim 1	2.43 (0.90)	5.51 (0.18)	3.83 (0.18)
TR-PTSD post-stim 2	2.37 (0.10)	5.56 (0.21)	3.66 (0.15)
TR-PTSD post-stim 3	2.39 (.13)	5.42 (0.26)	3.90 (0.22)
non-TR-PTSD	2.39 (0.13)	5.62 (0.28)	3.86 (0.17)
**Positive**	**Valence**	**Arousal**	**Dominance**
TR-PTSD pre-stim	7.70 (0.05)	5.47 (0.13)	6.23 (0.07)
TR-PTSD post-stim 1	7.58 (0.09)	5.07 (0.21)	6.18 (0.13)
TR-PTSD post-stim 2	7.56 (0.08)	5.12 (0.25)	6.12 (0.15)
TR-PTSD post-stim 3	7.55 (0.13)	4.97 (0.33)	6.20 (0.19)
non-TR-PTSD	7.73 (0.08)	5.13 (0.21)	6.29 (0.10)
**Neutral**	**Valence**	**Arousal**	**Dominance**
TR-PTSD pre-stim	4.97 (0.07)	3.48 (0.17)	5.67 (0.09)
TR-PTSD post-stim 1	5.12 (0.14)	3.56 (0.16)	5.80 (0.13)
TR-PTSD post-stim 2	5.11 (0.12)	3.55 (0.20)	5.82 (0.12)
TR-PTSD post-stim 3	5.10 (0.21)	3.70 (0.21)	5.73 (0.23)
non-TR-PTSD	5.79 (0.22)	3.90 (0.25)	5.92 (0.12)

Supplementary Table 6. Mean (s.e.m.) valence, arousal and dominance for IAPS emotional image sets used in the study for treatment-resistant post-traumatic stress disorder (TR-PTSD) and non-TR-PTSD (epilepsy) participants calculated from normalized ratings[2]. For TR-PTSD participants, ratings are separated for pre- (Pre-Stim) and post-stimulation (Post-Stim) sessions 1 and 2.

Negative	Brightness	Contrast	Entropy
TR-PTSD Pre-Stim	106.27 (6.27)	67.39 (2.14)	7.06 (0.18)
TR-PTSD Post-Stim 1	100.57 (7.14)	68.96 (3.00)	7.15 (0.22)
TR-PTSD Post-Stim 2	102.86 (7.92)	65.96 (3.24)	7.09 (0.23)
TR-PTSD Post-Stim 3	99.01 (8.49)	71.61 (3.78)	6.94 (0.25)
non-TR-PTSD	110.98 (8.45)	66.30 (3.65)	7.40 (0.10)
**Positive**	**Brightness**	**Contrast**	**Entropy**
TR-PTSD Pre-Stim	104.74 (7.22)	70.49 (2.74)	6.97 (0.19)
TR-PTSD Post-Stim 1	122.43 (8.27)	71.45 (3.49)	6.89 (0.22)
TR-PTSD Post-Stim 2	108.74 (7.93)	72.10 (2.68)	7.04 (0.19)
TR-PTSD Post-Stim 3	100.32 (7.03)	70.23 (2.72)	7.09 (0.21)
non-TR-PTSD	100.45 (5.98)	70.36 (2.64)	7.13 (0.17)
**Neutral**	**Brightness**	**Contrast**	**Entropy**
TR-PTSD Pre-Stim	116.89 (8.88)	64.16 (3.68)	6.95 (0.12)
TR-PTSD Post-Stim 1	108.81 (9.49)	63.14 (3.19)	6.89 (0.22)
TR-PTSD Post-Stim 2	102.8 (9.72)	66.05 (3.59)	7.04 (0.22)
TR-PTSD Post-Stim 3	101.15 (10.89)	60.04 (2.73)	6.69 (0.28)
non-TR-PTSD	95.45 (7.69)	60.65 (4.19)	7.05 (0.26)

Supplementary Table 7. **Psychophysical properties of emotional images.** Mean (s.e.m.) brightness, contrast and entropy for IAPS emotional image sets used in the study. Parameters calculated using previously described methods[3]. For TR-PTSD participants, ratings are separated for pre- (Pre-Stim) and post-stimulation (Post-Stim) 1 and 2 sessions.

Supplementary References

1. Marx, B. P. *et al.* Reliable and clinically significant change in the clinician-administered PTSD Scale for DSM-5 and PTSD Checklist for DSM-5 among male veterans. *Psychological Assessment* **34**, 197–203 (2022).
2. Lang, P. J., Bradley, M. M. & Cuthbert, B. N. International affective picture system (IAPS): Instruction manual and affective ratings, Technical Report A-8. *Gainesville: The center for research in psychophysiology, University of Florida* (2008).
3. Marchewka, A., Zurawski, Ł., Jednoróg, K. & Grabowska, A. The Nencki Affective Picture System (NAPS): introduction to a novel, standardized, wide-range, high-quality, realistic picture database. *Behav Res Methods* **46**, 596–610 (2014).
