# Supplementary material for: A pilot study of closed-loop neuromodulation for treatment-resistant post-traumatic stress disorder
Source: Nat Commun. 2023 May 24;14:2997. doi: 10.1038/s41467-023-38712-1 (PMC10209131; doi:10.1038/s41467-023-38712-1)
Supplement: Supplementary file 3 — Description of Additional Supplementary Files [file 41467_2023_38712_MOESM3_ESM.pdf]

Description of Additional Supplementary Files

File Name: Supplementary Video 1

Description: Simultaneous Emotional Image Task and iEEG activity from an example TR-PTSD participant during pre-stimulation.

Top, Example positive (blue), neutral (blue), and negative (red) International Affective Picture System images from the Emotional Image Task. **Middle**, Simultaneous normalized theta power from an example amygdala channel during pre-stimulus fixation (4s) and positive (blue), neutral (blue), and negative (red) images (4s each). **Bottom**, Same as the middle but showing the raw iEEG activity from the same amygdala channel. Flat lines represent times between selected trials and are for display purposes only.
