# Supplementary material for: A pilot study of closed-loop neuromodulation for treatment-resistant post-traumatic stress disorder
Source: Nat Commun. 2023 May 24;14:2997. doi: 10.1038/s41467-023-38712-1 (PMC10209131; doi:10.1038/s41467-023-38712-1)
Supplement: Supplementary file 5 — Reporting Summary [file 41467_2023_38712_MOESM5_ESM.pdf]

nature portfolio

Corresponding author(s): Nanthia Suthana, Jean-Philippe Langevin

Last updated by author(s): Apr 17, 2023

Reporting Summary

Nature Portfolio wishes to improve the reproducibility of the work that we publish. This form provides structure for consistency and transparency in reporting. For further information on Nature Portfolio policies, see our Editorial Policies and the Editorial Policy Checklist.

Statistics

For all statistical analyses, confirm that the following items are present in the figure legend, table legend, main text, or Methods section.

n/a	Confirmed	
☐	☒	The exact sample size (*n*) for each experimental group/condition, given as a discrete number and unit of measurement
☐	☒	A statement on whether measurements were taken from distinct samples or whether the same sample was measured repeatedly
☐	☒	The statistical test(s) used AND whether they are one- or two-sided<br>*Only common tests should be described solely by name; describe more complex techniques in the Methods section.*
☐	☒	A description of all covariates tested
☐	☒	A description of any assumptions or corrections, such as tests of normality and adjustment for multiple comparisons
☐	☒	A full description of the statistical parameters including central tendency (e.g. means) or other basic estimates (e.g. regression coefficient) AND variation (e.g. standard deviation) or associated estimates of uncertainty (e.g. confidence intervals)
☐	☒	For null hypothesis testing, the test statistic (e.g. *F*, *t*, *r*) with confidence intervals, effect sizes, degrees of freedom and *P* value noted<br>*Give P values as exact values whenever suitable.*
☒	☐	For Bayesian analysis, information on the choice of priors and Markov chain Monte Carlo settings
☒	☐	For hierarchical and complex designs, identification of the appropriate level for tests and full reporting of outcomes
☐	☒	Estimates of effect sizes (e.g. Cohen's *d*, Pearson's *r*), indicating how they were calculated

Our web collection on statistics for biologists contains articles on many of the points above.

Software and code

Policy information about availability of computer code

Data collection	Neuropace RNS System; Nihon Kohden continuous iEEG Monitoring
Data analysis	MATLAB 2021b (The MathWorks, Natick, MA, USA); Statistics and Machine Learning Toolbox for MATLAB 2021b; BOSC toolbox for MATLAB (eBOSC; version June 05, 2019), SPSS Statistics Software Package (IBM Corp. Released 2020), Freesurfer version 7.2.0.

For manuscripts utilizing custom algorithms or software that are central to the research but not yet described in published literature, software must be made available to editors and reviewers. We strongly encourage code deposition in a community repository (e.g. GitHub). See the Nature Portfolio guidelines for submitting code & software for further information.

Data

Policy information about availability of data

All manuscripts must include a data availability statement. This statement should provide the following information, where applicable:

- Accession codes, unique identifiers, or web links for publicly available datasets
- A description of any restrictions on data availability
- For clinical datasets or third party data, please ensure that the statement adheres to our policy

Data is available upon reasonable request

Human research participants

Policy information about studies involving human research participants and Sex and Gender in Research.

Reporting on sex and gender	The sex of all participants is reported. Sex was not factored into study design. Sex based analyses were not performed as all study participants were male.
Population characteristics	A total of 8 participants (all male, ages 28-68) with depth electrodes that were previously implanted for either treatment-resistant PTSD (TR-PTSD; N=2) or epilepsy (non-TR-PTSD, N=6) completed the study (Supplementary Table 1). More details can be found in Supplementary Tables 2 and 3 of the manuscript.
Recruitment	Treatment- resistant post traumatic stress disorder (TR-PTSD) participants were recruited from the Veteran Affairs Healthcare System (VAHS) and the Ronald Regan Medical UCLA Center (RRUMC). Referrals and recruitment were generated from chart review, treating psychiatrists, and other providers. All participants provided written, informed consent according to a protocol approved by the UCLA and VAHS Medical Institutional Review Boards (IRB). The supporting grant (UH3NS107673) limited recruitment to male participants, limiting generalization of finding to other sexes. Our sample is also biased towards combat veterans given the use of VAHS as the major recruitment site, potentially limiting findings to a specific subgroup of the PTSD population.<br><br>Non-TR-PTSD participants were referred by treating physicians at UCLA and VAHS following expression of interest in research participation.
Ethics oversight	All participants volunteered for the study by providing informed consent according to a protocol approved by the UCLA and VAHS Medical Institutional Review Boards.

Note that full information on the approval of the study protocol must also be provided in the manuscript.

Field-specific reporting

Please select the one below that is the best fit for your research. If you are not sure, read the appropriate sections before making your selection.

[x] Life sciences [ ] Behavioural & social sciences [ ] Ecological, evolutionary & environmental sciences

For a reference copy of the document with all sections, see nature.com/documents/nr-reporting-summary-flat.pdf

Life sciences study design

All studies must disclose on these points even when the disclosure is negative.

Sample size	A total of 8 participants (all male, ages 28-68) with depth electrodes that were previously implanted for either treatment-resistant PTSD (TR-PTSD; N=2) or epilepsy (non-TR-PTSD, N=6) completed the study. TR-PTSD participants were diagnosed with severe, chronic combat PTSD (CAPS-5 score > 47 across > 8 weeks, total illness duration ≥ 5 years with no period of clinical remission), and clinically significant impairment in social and occupational functioning due to PTSD (≥ 70% service-connected disability, Global Assessment of Functioning Score (GAF)16 ≤ 45, or no period of full-time employment for longer than 3 months in the past 5 years). Their PTSD symptoms corresponded to stage 2 of treatment-resistance for PTSD.17 Clinical record documented failure to respond to adequate (minimum 3 month, with adherence) trials of at least 3 evidence-based treatments including at least one pharmacologic agent (sertraline, paroxetine, fluoxetine or venlafaxine), and at least one trauma-focused individual cognitive-behavioral psychotherapy (either Prolonged Exposure Therapy [PE], Cognitive Processing Therapy [CPT], Eye movement Desensitization and Reprocessing [EMDR], or other form of evidence-based cognitive behavioral therapy for PTSD). TR-PTSD participants were chronically implanted with the RNS system (NeuroPace, Inc., Mountain View, CA) as part of a clinical trial (ClinicalTrials.gov [NCT04152993]), whereas epilepsy participants were implanted during clinical care, three with acute stereoelectroencephalography (sEEG) electrodes for evaluation of pharmaco-resistant epilepsy and three with a chronically implanted RNS System (NeuroPace, Inc., Mountain View). Given the rare opportunity to test with such patients, the sample size was informed by availability for and interest in study participation. Though limited, we believe that this initial pilot investigation provides reasonable support for discussed findings.
Data exclusions	Across non-TR-PTSD participants electrodes were in a variety of structures including the amygdala, hippocampus, prefrontal cortex, and cingulate cortex. Only electrodes located within the amygdala (n=10) contributed to non-TR-PTSD analyses to match analyses completed in TR-PTSD participants in whom four channels were in the amygdala.
Replication	The emotional image task was performed eight times independently with eight different participants. The task was repeated three times at three separate time points (Post-Stim 1-3) in TR-PTSD. The script driven imagery task was repeated three times (Post-Stim 1-3) in two different TR-PTSD participants. As described in the study, response to task stimuli are related to clinical response to closed loop therapy.<br><br>Separate investigations in TR-PTSD participants will be necessary to replicate results All methods used to perform this study and analyses needed to replicate the presented findings are detailed in the Methods section of the manuscript. Data were statistically analyzed on the group-level (i.e., non-TR-PTSD and TR-PTSD). Though further investigations are necessary to characterize differences between TR-PTSD and non-TR-PTSD neural dynamics related to emotional stimulus processing, the compelling group differences reported provide a necessary first step.

Randomization	All participants were tested with the same experimental protocol. They were not assigned to different experimental groups.
Blinding	All experimenters were aware and not blinded with regards to the participant's condition (i.e. TR-PTSD, pharmaco-resistant epilepsy).

Reporting for specific materials, systems and methods

We require information from authors about some types of materials, experimental systems and methods used in many studies. Here, indicate whether each material, system or method listed is relevant to your study. If you are not sure if a list item applies to your research, read the appropriate section before selecting a response.

Materials & experimental systems

n/a	Involved in the study
☒	☐ Antibodies
☒	☐ Eukaryotic cell lines
☒	☐ Palaeontology and archaeology
☒	☐ Animals and other organisms
☐	☒ Clinical data
☒	☐ Dual use research of concern

Methods

n/a	Involved in the study
☒	☐ ChIP-seq
☒	☐ Flow cytometry
☐	☒ MRI-based neuroimaging

Clinical data

Policy information about clinical studies

All manuscripts should comply with the ICMJEguidelines for publication of clinical research and a completedCONSORT checklist must be included with all submissions.

Clinical trial registration	ClinicalTrials.gov NCT04152993
Study protocol	A full study protocol is attached with supporting documentation for the manuscript and can also be found at ClinicalTrials.gov NCT04152993
Data collection	PTSD symptom severity was tracked in both TR-PTSD participants using the Clinician-Administered PTSD Scale for DMS-5 (CAPS-5)13. Assessment interviews were administered and rated by the study psychiatrist (R.J.K.) and clinical psychologist (J.S.) using the CAPS-5 which reflects disease status during the preceding month. CAPS-5 scores were collected 5-6 months and 1 month before surgery, 1 month after surgery (before stimulation initiation) and 2, 3, and 4 months post stimulation for a total of 6 measures in each TR-PTSD participant. Clinical data was collected via video chat (due to COVID precautions) and in person at the medical facility of the treating physician. Both TR-PTSD participants were recruited in the same year and began the study protocol one month apart from one another.
Outcomes	Prior to participant enrollment, the primary outcome of the clinical trial was defined as 12 months of Clinician Administered PTSD Scale (CAPS) monitoring. To assess the magnitude of individual PTSD symptom change due to stimulation, we computed difference scores of mean CAPS-5 scores across assessments before and after stimulation onset for each participant. Symptom changes were classified as indicative of reliable change when the difference scores were greater than the threshold for identifying clinically meaningful change for male combat veterans reported by Marx et al (Reference 14).

Magnetic resonance imaging

Experimental design

Design type	MRI was used only to determine the localization of electrode contacts within the brain.
Design specifications	MRI was used only for electrode contact localization; thus, participants did not perform an experimental task during MRI scanning.
Behavioral performance measures	No behavioral performance measures were acquired or derived, since participants did not perform an experimental task during MRI scanning.

Acquisition

Imaging type(s)	Structural
Field strength	3 Tesla
Sequence & imaging parameters	Standard T1- and T2-weighted sequences
Area of acquisition	Whole-Brain

Diffusion MRI ☐ Used ☒ Not used

Preprocessing

Preprocessing software	MRI data were preprocessed using FSL (FMRIB Software Library, Oxford University, UK; v5.0.11) for image registration with the FLIRT function (default parameters), and ITK-SNAP (version 3.8.0) for visualization and manual segmentation of electrode contacts.
Normalization	All MRI images were registered to standardized space.
Normalization template	MNI152_T1_2mm_brain.nii
Noise and artifact removal	No noise or artifact removal procedures were applied.
Volume censoring	Volume censoring was not applied.

Statistical modeling & inference

Model type and settings	No model-based analyses were performed using MRI data.
Effect(s) tested	MRI was used only for electrode contact localization; thus, no task- or stimulus-related analyses were performed using MRI data.

Specify type of analysis: [x] Whole brain [ ] ROI-based [ ] Both

Statistic type for inference (See Eklund et al. 2016)	No statistical analyses were performed using MRI data.
Correction	No statistical analyses were performed using MRI data; thus, no correction methods were applied.

Models & analysis

n/a	Involved in the study
[x]	[ ] Functional and/or effective connectivity
[x]	[ ] Graph analysis
[x]	[ ] Multivariate modeling or predictive analysis
